# Intelligent algorithm based on deep learning to predict the dosage for anesthesia: A study on prediction of drug efficacy based on deep learning

**DOI:** 10.1002/hsr2.2113

**Published:** 2024-05-14

**Authors:** ZhiGang Hu, GuangJian Pan, XinZheng Wang, KeHan Li

**Affiliations:** ^1^ Department of Biomedical Engineering Henan University of Science and Technology Luoyang China; ^2^ Department of Anesthesiology The First Affiliated Hospital of Henan University of Science and Technology LuoYang China

**Keywords:** anesthesia dose prediction, attention mechanism, deep learning

## Abstract

**Background and Aims:**

Anesthetic drugs play a vital role during surgery, however, due to individual differences and complex physiological mechanisms, the prediction of anesthetic drug dosage has always been a challenging problem. In this study, we propose a model for predicting the dosage of anesthetic drugs based on deep learning to help anesthesiologists better control their dosage during surgical procedures.

**Methods:**

We design a model based on the artificial neural network to predict the dosage of preoperative anesthetic, and use the SELU activation function and the loss function for weighted regularization to solve the problem of unbalanced sample. Moreover, we design a CNN‐based model for the prior extraction of intraoperative features by using a 7 × 1 convolution kernel to enhance the receptive field, and combine maximum pooling and average pooling to extract key features while eliminating noise. A predictive model based on the LSTM network is designed to predict the intraoperative dosage of the anesthetic, and the bidirectional propagation‐based LSTM network is used to improve the ability to learn the trend of changes in the physiological states of the patient during surgery. An attention module is added before the connection layer to appropriately attend to areas containing prominent features.

**Results:**

The results of experiments showed that the proposed method reduced values of the MAPE to 15.83% and 12.25% compared with the traditional method in predictions of the preoperative and intraoperative doses of the anesthetic, respectively, and increased the values of $R^{2}$ to 0.887 and 0.915, respectively.

**Conclusion:**

The intelligent anesthesia prediction algorithm designed in this study can effectively predict the dosage of anesthetic drugs needed by patients, assist clinical judgment of anesthetic drug dose, and assist the anesthesiologists to ensure the smooth progress of the operation.

## 1 INTRODUCTION

In the field of medicine, anesthesiology is a long‐established and critical discipline. Since ancient times, mankind's pursuit of painless surgery and comfortable medical treatment has given rise to the birth and development of anesthesiology. From the initial ether anesthesia to modern anesthesia methods such as general anesthesia, local anesthesia and nerve block, anesthesiology continues to make breakthroughs in technological innovation and clinical practice.^1^ However, precise control of anesthetic drug dosage remains a challenging issue due to factors such as individual differences, type of surgery, patient age, and weight. An excessive dose may cause the patient to suffer from serious complications like respiratory depression and cardiovascular system depression, while an insufficient dose may lead to the patient becoming conscious and experiencing intraoperative pain and memory. Therefore, accurately predicting anesthetic drug dosage has crucial clinical significance.^2^, ^3^ With the rapid advancement of science and technology, particularly the rise of artificial intelligence technology, intelligent anesthesia has become the new favorite of modern medicine, injecting new vitality into the development of anesthesiology.

However, the development of smart anesthesia has faced significant challenges. First of all, traditionally, manually adjusting anesthetic doses is heavily influenced by the clinician's experience and subjective judgment, leading to inaccurate dose control.^1^ Therefore, improving surgical efficiency while ensuring patient safety is crucial for enhancing medical standards and reducing the risk of medical accidents. Secondly, the practical application of intelligent anesthesia systems presents several challenges. Ensuring the accuracy and reliability of these systems is paramount. Balancing the automation of intelligent anesthesia with the professional judgment of physicians is another key consideration. Achieving widespread adoption of intelligent anesthesia while prioritizing patient safety requires in‐depth research and discussion^4^, ^5^

Anesthesiologists determine the doses of anesthetic drugs for patients during surgery based on their clinical experience because their mechanism of action is not fully understood, and no universal standard is available for their administration. A number of studies have shown that such indicators of physiological health as the heart rate (HR), electrocardiograph (ECG) signals, pulse waves (photoplethysmography, PPG), and the bispectral index of the electroencephalogram can reflect the influence of drugs on the central nervous system. They represent the patient's state of consciousness^6^, ^7^ and can be used to predict the dose of the anesthetic for the patient. Prevalent studies have sought to predict the depth of anesthesia in patients during surgery based on ECG and PPG signals, and have used this to determine the appropriate dose of anesthetic drugs. On this basis, Zhang, W.W. et al. utilized the permutation and entropy algorithms to detect the depth of anesthesia in patients by extracting the characteristic parameters of different fields of EEG signals.^8^ However, relying solely on EEG signals may not fully capture the patient's physiological characteristics. To address this, Roy Chowdhury, M. et al.^9^ proposed a method to predict anesthesia depth based on ECG and PPG signals. They segmented one‐dimensional (1D) ECG and PPG signals into 2D images, and used a multi‐layer convolutional neural network^10^ (CNN) to predict the depth of anesthesia. Based on previous research on ECG and PPG, Guo, Y. et al. added heart rate variability (HRV) as input. They used the transfer learning, and implemented anesthesia depth classification based on artificial neural networks.^11^ The results of experiments showed that it can achieve an accuracy of classification of 94.1%. There is still room for improvement in the prediction effect of anesthesia depth prediction based on ECG, PPG, and HRV based on traditional methods and machine learning methods. Therefore, Dutt, M.I. et al. proposed an accurate method of LoH estimation based on the stationary wavelet transform and fractal features.^12^ They used optimized sets of temporal, fractal, and spectral features to identify the level of sedation of patients. The results of experiments showed that the LoH classifier proposed by Dutt et al. outperformed state‐of‐the‐art algorithms for LoH prediction, and attained a highest accuracy of 97.1% when using the minimum feature set and the MLP classifier.

But traditional anesthesia administration relies on the doctor's experience and judgment, and there is the possibility of human error. However, the accuracy of anesthesia delivery based on machine learning has not yet reached the ideal effect. The intelligent anesthesia administration algorithm based on deep learning can reduce this kind of error. Compared with machine learning, anesthesia treatment is more accurate and reliable. In addition, deep learning algorithms can implement personalized anesthesia dosing plans based on patients’ individual differences and changes in condition. Each patient's physiological condition, metabolic rate, drug response and other factors are different. Intelligent algorithms can adjust the dose and administration rate of anesthetics based on this information to ensure the best therapeutic effect and safety for the patient.

Based on the prediction of anesthesia depth, researchers began to study the prediction model of anesthetic drug dosage. For example, Nanaka et al. built a model to predict the optimal dose of anesthesia‐inducing drugs by focusing on electronic anesthesia records and using a regression model, one of the machine learning methods. By adjusting the explanatory variables and parameters and using ridge regression, the determined prediction coefficient was only 0.5008,^13^ so there is still a lot of room for improvement in the accuracy of anesthetic drug dose prediction. Therefore, based on previous research on anesthesia depth prediction and anesthetic drug dose prediction, this article proposes the use of deep learning methods, fully considering the timing of various physiological indicators of patients during surgery, and accurately and efficiently predicting the patient's anesthetic dose during surgery. According to the surgery Various physiological information of the patient during the process can be used to determine the patient's current anesthesia status, automatically adjust the anesthetic dose, improve the anesthesia effect and safety, and reduce patient risks and the anesthesiologist's work pressure.

Among them, the specific contributions of this article are as follows:

1. Collect patient clinical data, conduct preliminary screening of features through statistical analysis and recursive feature elimination,^14^ select the optimal feature combination as the input of the prediction model, and finally create a data set for use in this research. Among them, the constructed data set is divided into a training data set and a verification data set.
2. Design a preoperative anesthesia prediction model based on artificial neural network (ANN)^15^ and an intraoperative anesthesia prediction model based on CNN‐Long short‐term memory (LSTM),^16^ and use the training data set to train the network model.
3. After the prediction model training is completed, use the validation data set to verify and evaluate the model, ensuring that the model achieves the expected prediction performance.

## 2 METHOD

### 2.1 Data collection procedure

We used data on anesthesia provided by the Department of Anesthesiology of The First Affiliated Hospital of Henan University of Science and Technology. We referred to the literature^17^ and developed inclusion and exclusion criteria. The inclusion criteria were that patients met the following conditions at the time of enrollment: (1) age at least 5 years old, (2) no history of allergy to anesthetic drugs, and (3) having complete surgical records. The exclusion criteria were if they (1) had a history of allergy to anesthetic drugs, (2) were younger than 5 years old or older than 90 years old, or (3) had incomplete surgical records. These participants should all be excluded.

In total, 202 data points were compiled, including 68 samples from male patients and 134 from female patients. The ages of the patients ranged from 5 to 75 years, their weights from 10 to 85 kg, and the duration of the operations was 134 ± 34 min. The specific information of the sample data is as follows: The data include the patient's various physiological signs information and the dosage of various drugs during the entire operation. Physiological sign information that may affect the dosage of anesthetic drugs was collected and organized, and significance testing and feature recursive elimination methods were used to eliminate them, retaining only highly significant variables as input to the prediction model. And then we sorted all the sample data into two sets: preoperative and intraoperative data. The input features for the preoperative data set included information on the gender, age, weight, diastolic blood pressure (DBP), and systolic blood pressure (SBP) of the patients, and the output labels were the doses of three anesthetic drugs used for sedation, analgesia, and muscle relaxation. The input features for the intraoperative data set included the periodic records of surgery (recorded every 5 min), DBP and SBP, and the output label consisted of the type and dosage of the anesthetic administered to the patient during the operation. The data set was divided into training and validation data sets. The training data set was used to train the prediction model, and the validation data set was used to evaluate the model's prediction effect, using evaluation indicators such as MAE, MSE, and MAPE to assess the prediction accuracy.

### 2.2 Data processing

**Figure 1:**
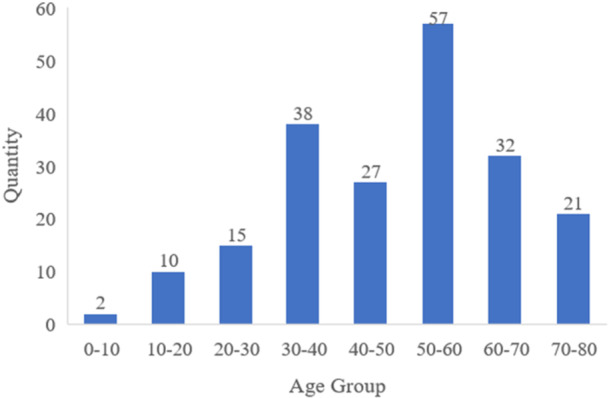
Data set sample distribution.

We organize the collected data samples and make full use of the data information in each sample. To make the model in this article have higher generalization ability, this article uses some missing data to avoid overfitting of the model and ensure that the performance of the model achieves the desired effect. The distribution of patients from each age group in the original data set is shown in Figure 1, which reveals an uneven distribution that does not conform to a normal distribution. Furthermore, the data set contained missing data, and the overall volume was insufficient. Consequently, we processed the original data set to generate an enriched data set. While ensuring data reliability, it improves the prediction accuracy and generalization ability of the model.

Some key features were missing from the data on the patients. We used the K‐nearest neighbors (KNN) method^18^ to fill in the missing information. It identified several items of data that were the closest to the missing data, and used their average value to replace the missing values.

Because the duration of each operation for each patient was different, the volume of data collected for each patient was different as well. We amplified the information on patients for whom the volume of available data was insufficient. We calculated the average value of two consecutive records as the new data item and inserted it into the data set to ensure that the volume of data for each patient was consistent, while ensuring that the trend of changes in various physiological indicators remained unchanged.

There were linear correlations between certain features in the curated data. Adding them directly to the model to train it would have increased the amount of requisite calculation while increasing the size of the model such that it would have yielded subpar predictions. We thus used recursive feature elimination to eliminate redundancy between the features before sending them to the model to train it, and obtained the optimal combination of features.

Following the above operations for data preprocessing, we filled in missing values in the data set to increase its size while ensuring that the original data remained unchanged. We also chose the optimal combination of features from among the input features and sent them to the network to train the model and improve its predictive accuracy.

### 2.3 Statistical analysis

We received a total of 234 pieces of data. After eliminating incomplete questionnaires, exploratory factor analysis was conducted on the 202 complete questionnaire data sets using IBM SPSS 28.0. The purpose of exploratory factor analysis is to uncover underlying factors and reduce dimensionality by grouping items into one or more latent variables. Its main variables include characteristics such as age, gender and weight in the data. When conducting single‐factor variable analysis, except for the gender group, which is divided into male and female groups, the remaining continuous variables are grouped using the median as the critical value to ensure a balance in the number of data samples in each group. Calculate 95% confidence intervals (CI) and two‐sided p values. A p value less than 0.05 was considered statistically significant. As shown in Table 1, the significance level of gender, age, and weight is significant, the significance level of systolic blood pressure and diastolic blood pressure is significant, and the significance level of BIS, pulse, etc. is not significant. Therefore, factors affecting model fitting include gender, age, weight, systolic blood pressure, and diastolic blood pressure, which is consistent with our previous results using the recursive feature elimination.

**Table 1:** Statistical relationship between physiological indicators and anesthetic drug dosage.

Variable		Frequency		*p*
sex	male	103	50.99%	0.01
	female	99	49.01%	
age	<53	98	48.51%	0.02
	≥53	104	51.49%	
weight	<82	100	49.50%	<0.01
	≥82	102	50.50%	
diastolic blood pressure	<128	101	50.00%	0.03
	≥128	101	50.00%	
systolic blood pressure	<86	104	51.49%	0.04
	≥86	98	48.51%	
BIS	<85	100	49.50%	>0.05
	≥85	102	50.50%	
pulse	<75	46	22.28%	>0.05
	≥75	156	77.72%	

### 2.4 Ethical consideration

This original study was approved by the research Ethics Committee of The First Affiliated Hospital of Henan University of Science and Technology and did not require informed consent from participants. The identities of participants were kept anonymous, with each being assigned a numerical identifier. All identifiers were removed and the data were stored in an encrypted, password‐protected file.

### 2.5 Proposed method

**Figure 2:**
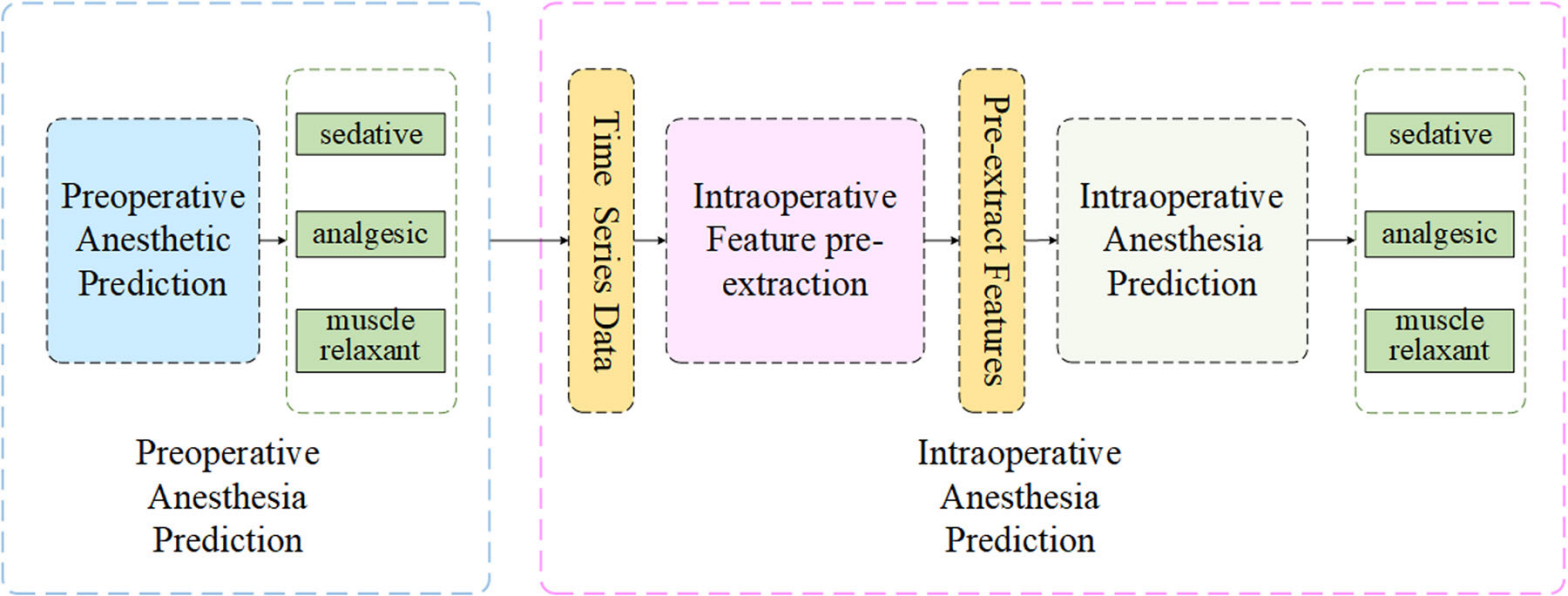
ANN‐CNN‐LSTM model structure.

We examined the procedures used for anesthesia during the entire operation, and used the information obtained to design the network model shown in Figure 2. The model is divided into three modules: those for predicting the dose of the preoperative anesthetic, extracting the intraoperative features beforehand, and predicting the dose of the intraoperative anesthetic.

The module to predict the dose of the preoperative anesthetic was designed based on the ANN. It received the initial physiological indicators before the operation as input, and its output layer provided the predicted preoperative dose of the anesthetic. The module to extract intraoperative features beforehand was based on the CNN. It received various physiological indicators of the patient under anesthesia as the input and output their feature values. The module to predict the dose of the intraoperative anesthetic was based on the LSTM network. It received the feature values output of the module to extract intraoperative features as the input. They were passed through two layers of the LSTM network and an attention network, following which the module generated the predicted dose of the intraoperative anesthetic.

#### 2.5.1 Predicting the dose of the preoperative anesthetic

Before the operation, a certain dose of anesthetic drugs needs to be administered to the patient. The volume of the dose is determined by the anesthesiologist based on their clinical experience. To reduce the burden on them and improve the accuracy of administration of this dose, we design a module to predict the dose of the preoperative anesthetic.

**Figure 3:**
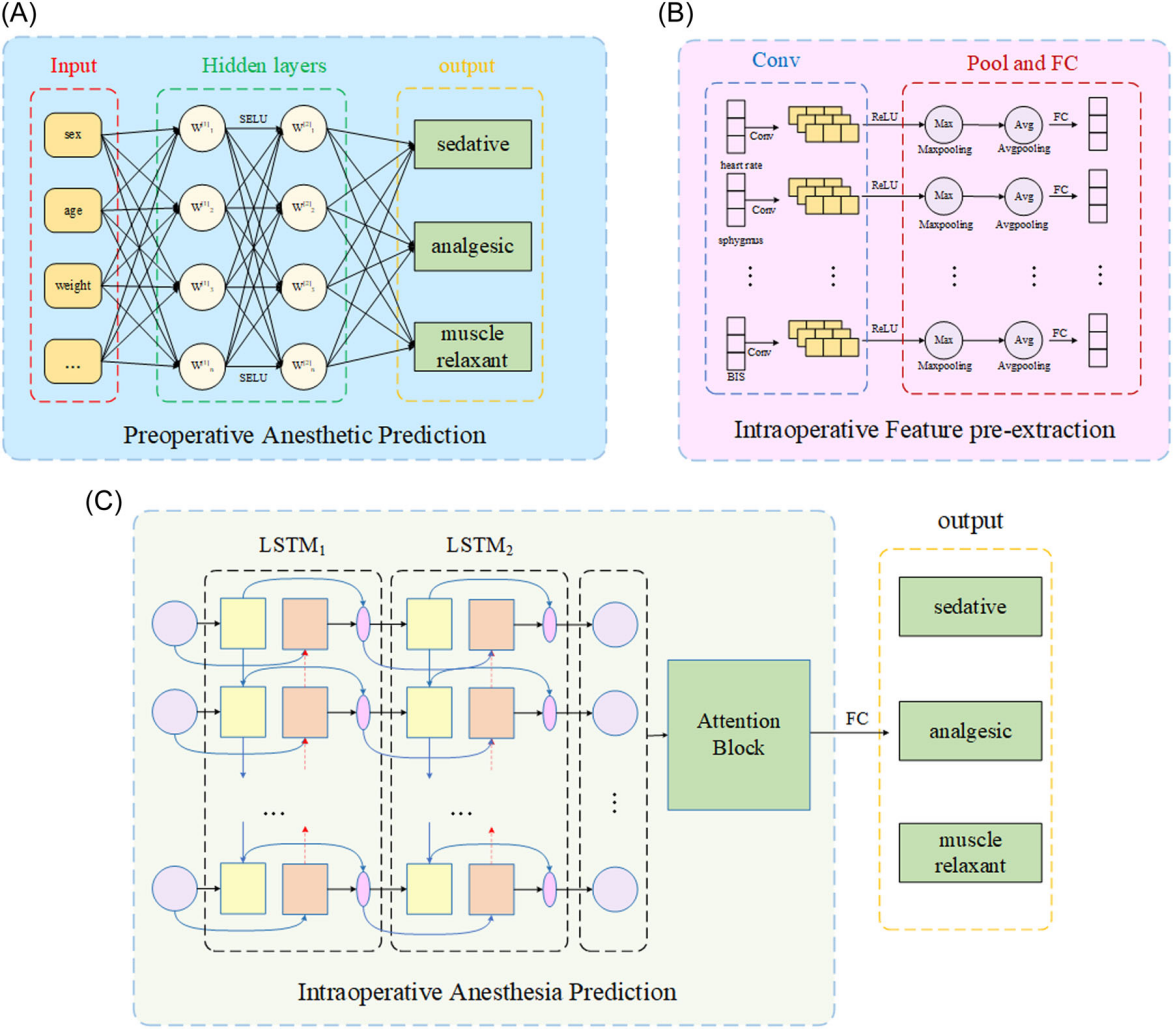
(A) Preoperative anesthesia prediction network structure, (B) Intraoperative feature extraction network structure, (C) Intraoperative anesthesia prediction network structure.

The module predicts the doses of various anesthetic drugs before the operation according to the physiological indicators of the patient. It is a multi‐input multi‐output model in which the relationship between the input and the output is nonlinear. The ANN network with excellent capability of fitting is used to design this module, and its structure is shown in Figure 3A It contains an input layer, two hidden layers, a fully connected layer, and an output layer.

The basic ANN cannot obtain suitably accurate predictions on the data set used in this paper. Considering the problem of an imbalance in the number of samples, and given that they did not obey the normal distribution, we used the SELU activation function,^19^ as shown in Formula (1). Once each neuron had passed through the activation function, the sample distribution was automatically normalized to that with a zero mean and unit variance. The input data could then be adaptively normalized to eliminate the non‐normal distribution of the output of the activation function in the neural network. This also caused the network to converge more quickly and improved the predictive accuracy of the model:

(1) $$\begin{array}{c} SeLU(x)=\left\{\begin{array}{ll} x & \text{if}\;x>0\\ \alpha e^{x}-\alpha & \text{if}\;x\leq 0 \end{array}\right.\\ \lambda \;=\;1.050700987355480493419334985294\\ \alpha \;=\;1.673263242354377284817042991671 \end{array}$$

We calculated the weighted loss during training, as shown in Formula (2), to further solve the problem of imbalance in the samples by increasing the weights of positive and negative samples. A regularization‐based penalty function was also added, as shown in Formula (3). The complexity of the model was controlled by applying a penalty to the sum of the squares of its parameters to prevent overfitting and improve its capability of generalization.

(2) $$J(\theta )=\sum_{i=1}^{m}{\left(h_{\theta }(x_{i})-y_{i}\right)}^{2}*\frac{m}{m_{c_{y_{i}}}}$$

(3) $$L(\theta )=J(\theta )+\lambda �p\;{�}_{2}^{2}$$

Where m is the total number of samples, $m_{c_{y_{i}}}$ is the number of samples of patients in the age group to which sample $y_{i}$ belongs, $h_{\theta }(x_{i})$ is the output of the model, λ is the regularization coefficient, and p represents all parameters of the model.

#### 2.5.2 Intraoperative feature extraction

The module used to extract intraoperative features in advance is designed based on the CNN, and its structure is shown in Figure 3B. It includes an input layer, three convolutional layers, two pooling layers, a fully connected layer, and an output layer.

Because the input to the network consists of time series data, we needed to identify the trends of changes in various physiological indicators of the patients under anesthesia. As the CNN has excellent capabilities of feature extraction, changing the size of its convolution kernel can yield receptive fields of different sizes. We thus replaced the original 3 × 1 small convolution kernel with a larger 7 × 1 convolution kernel to obtain a larger receptive field and better features over a longer period.

The pooling layer uses a combination of maximum pooling and average pooling to further extract the global features. Maximum pooling can eliminate noise and useless information, while average pooling extracts and retains the overall characteristics of the data, and identifies changes in various physiological indicators over a long period. The combination of maximum pooling and average pooling can prevent overfitting of the model and improve its robustness while extracting the characteristics of the physiological indicators.

#### 2.5.3 Predicting the dose of the intraoperative anesthetic

The module used to predict the intraoperative dose of the anesthetic does so based on various physiological indicators of the patient during the operation, and determines whether a second dose of anesthetics is needed based on changes in these indicators. It provides the recommended dosage of secondary anesthetic drugs if the patient is about to wake up during the operation. As the physiological characteristics of the patient change over time during the operation, the module for predicting the dose of the intraoperative anesthetic is designed using the LSTM network and its structure is shown in Figure 3C. The module contains an input layer, two LSTM units, an attention module, a fully connected layer, and an output layer.

The LSTM layer uses the physiological features extracted by the module used to extract intraoperative features in advance as the input, and obtains the historical physiological information on the patient to better represent changes in the global features through bidirectional propagation to train the network. Forward propagation and backpropagation can be used to predict the trend of changes in the patient's physiological information during the training of the model. The bidirectional propagation‐based LSTM network can extract the global characteristics of various physiological indicators to improve the accuracy of the model.

To enable the network to pay more attention to changes in the physiological state of the patient from the state of anesthesia to recovery and refine the extracted features, we add an attention module before the fully connected layer. The attention mechanism can also help solve such problems as gradient disappearance and explosion, and can improve the speed of training and predictive accuracy of the model.

## 3 RESULT

### 3.1 Evaluation criteria

We used the mean absolute error (MAE), mean squared error (MSE), mean absolute percentage error (MAPE), and coefficient of determination as the criteria to assess the predictive model:

(4) $$MAE\left(y,\hat{y}\right)=\frac{1}{m}\sum_{i=1}^{m}\left(|y_{i}-{\hat{y}}_{i}|\right)\;$$

(5) $$MSE\left(y,\hat{y}\right)=\frac{1}{m}\sum_{i=1}^{m}{\left(|y_{i}-{\hat{y}}_{i}|\right)}^{2}$$

(6) $$MAPE(y,\hat{y})=\frac{1}{m}\sum_{i=1}^{m}\frac{\left|y_{i}-{\hat{y}}_{i}\right|}{max(\varepsilon ,|y_{i}|)}$$

(7) $$R^{2}(y,\hat{y})=1-\frac{\sum_{i=1}^{m}{\left(y_{i}-{\hat{y}}_{i}\right)}^{2}}{\sum_{i=1}^{m}{\left(y_{i}-\bar{y}\right)}^{2}}$$

where $y_{i}$ is the real value of the given sample, ${\hat{y}}_{i}$ is the output predicted by the model, and $\bar{y}$ is the average value of y. The MAE calculates the absolute value of the difference between the predicted value and the real value of each sample, and then sums and averages the differences for all samples. The MSE calculates the square of the difference between the predicted value and the true value of each sample, and then sums and averages the differences for all samples. The MAPE is the percentage of absolute error between the true and the predicted values. ε is an arbitrarily small positive number used to avoid undefined results when $y_{i}$ is zero. The coefficient of determination $R^{2}$ measures the ratio of variations in the dependent variable that can be explained by the independent variable to judge the explanatory power of the statistical model. Its value ranges from zero to one. The closer $R^{2}$ is to one, the better is the performance of the model, and vice versa. A negative value of $R^{2}$ indicates very poor performance.

### 3.2 Results and analysis

We used datasets of anesthesia obtained from the First Affiliated Hospital of Henan University of Science and Technology to evaluate the predictions of the proposed algorithm. All experiments were carried out on the Windows GPU computing platform, with a CPU Intel Core i9 12900KF, 32 GB memory, an NVIDIA GTX3060Ti GPU, and an Intel®c612 chipset. All programs were implemented on the open‐source framework PyTorch.

We used fivefold cross‐validation for the evaluation. The data set was divided into five equal parts, and one part was used as the test set and the other four parts as the training set. The average value obtained over four tests was used to evaluate the predictions of the proposed method in comparison with commonly used methods in the area. We needed to select the appropriate hyper‐parameters before training, including the batch size and the learning rate. The batch size was chosen by considering the capacity for video memory, and its maximum value was set to 32. The learning rate was determined by the curve of error of the model during training. If the curve drops slowly, the learning rate should be increased, while if the curve oscillates significantly, the learning rate should be reduced. We set the learning rate to 0.001 throughout the experiment to ensure the stable convergence of the model.

#### 3.2.1 Model optimization

We used the SELU function, which is self‐normalizable, as the activation function to solve the problem of unbalanced samples in the data set. It can converge to a mean value of zero, a variance of one, or a variance with upper and lower bounds even in the presence of noise. A comparison of the results of models that used different activation functions based on different evaluation indicators is shown in Table 2. It is clear that the model incurred the smallest loss in the initial stage of training and yielded the highest value of $R^{2}$ when using the SELU activation function. The SELU activation function could thus improve the accuracy of predictions by the model.

**Table 2:** Comparison of prediction results of different activation functions.

Activation functions	Evaluation criteria			
	MAE	MSE	MAPE	R2
ReLU	3.15	14.76	17.59	0.856
Tanhshrink	4.91	16.32	18.72	0.798
Sigmoid	3.36	15.88	16.32	0.833
**SELU**	**2.62**	**12.86**	**15.83**	**0.887**

We compared the influence of four convolution kernels on the performance of the module used to extract intraoperative features in advance, and the results are shown in Table 3. It is clear that as the size of the convolution kernel increased, the predictions of the module became more accurate. However, when the convolution kernel was larger than 7 × 1, the MSE of the model increased, and it became prone to overfitting. It thus yielded poor predictions. We thus used a 7 × 1 convolution kernel in the module to extract intraoperative features in advance to improve the predictive accuracy and robustness of the entire model.

**Table 3:** Comparison of prediction results of convolution kernels of different sizes.

Kernel size	Evaluation criteria			
	MAE	MSE	MAPE	R2
3*1	3.11	13.68	13.26	0.856
5*1	2.67	15.43	12.74	0.884
7*1	**2.13**	**10.25**	**12.25**	**0.915**
9*1	2.35	10.54	12.89	0.891

#### 3.2.2 Predictions of the overall model

Based on the prediction of anesthetic drug dosage during the entire surgical process, we proposed an ANN‐CNN‐LSTM prediction model. But the entire surgical process is divided into two processes, preoperative and intraoperative, so we will discuss the two processes separately: the preoperative anesthetic drug dose prediction model based on ANN and the intraoperative anesthetic drug dose prediction model based on CNN‐LSTM. The specific comparison experiments are as follows:

We compared predictions of the proposed model with those of lasso regression,^20^ polynomial regression,^21^ and the GRNN^22^ on the same data set to assess its performance. The results are shown in Table 4. (Preoperative anesthetic drug prediction).

**Table 4:** Comparison of anesthetic prediction results.

Models	Evaluation criteria			
	MAE	MSE	MAPE	R2
**Preoperative anesthetic drug prediction**				
Lasso Regression	3.16	16.35	18.8	0.792
Polynomial Regression	2.81	12.95	16.88	0.863
GRNN	2.74	13.73	16.54	0.856
**Our Improved ANN**	**2.62**	**12.86**	**15.83**	**0.887**
**Intraoperative anesthetic drug prediction**				
RNN	3.86	16.85	16.37	0.833
LSTM	3.43	12.35	15.58	0.869
LSTM‐Attention	2.96	11.59	13.69	0.873
CNN‐LSTM	2.54	11.32	12.57	0.881
**CNN‐LSTM‐Attention**	**2.13**	**10.25**	**12.25**	**0.915**

Table 4 shows that errors incurred by the proposed model were smaller than those of the other methods. It recorded an MAPE of 15.83%, which was 2.97%, 1.05%, and 0.71% smaller than those of lasso regression, polynomial regression, and the GRNN, respectively. Its value of $R^{2}$ was 0.887, an increase of 9.5%, 2.4%, and 3.1% over the values obtained by lasso regression, polynomial regression, and the GRNN, respectively.

We also compared our proposed model to predict doses of the anesthetic with LSTM‐Attention and CNN‐LSTM, and the results are shown in Table 4. (Intraoperative anesthetic drug prediction) The proposed method recorded an MAPE of 12.25%, which was 4.12%, 3.33%, 1.44%, and 0.32% lower than those of the RNN, LSTM, LSTM‐Attention, and CNN‐LSTM, respectively. In addition, its value of $R^{2}$ was 0.915, which was 8.2%, 4.6%, 4.2%, and 3.4% higher than those of the RNN, LSTM, LSTM‐Attention, and CNN‐LSTM, respectively. These results show that the proposed model, equipped with the modules to extract preoperative features and the attention mechanism, yielded the best predictions.

## 4 DISCUSSION

Anesthetic drug dose prediction based on deep learning is an important research area, which is crucial to ensure the safety of surgical procedures and patient comfort. By accurately predicting the dosage of anesthetic drugs, physicians can better control the patient's depth of anesthesia, reduce adverse reactions, and improve the success rate of surgery. In this paper, we propose a prediction model that combines ANN, CNN, and LSTM to achieve accurate prediction of anesthetic drug dosage throughout the surgical procedure.

First, we discuss preoperative anesthetic dose prediction models. At this stage, we mainly rely on static data such as patients’ personal information. We utilize the powerful nonlinear mapping capabilities of ANN to build a model that can learn and simulate complex relationships. By inputting these static data, ANN can predict the dosage of anesthetic drugs required before surgery, providing an initial guarantee for the safety of the surgery.

Next, we focus on the intraoperative anesthetic dose prediction model. Different from the preoperative stage, the intraoperative stage involves more dynamically changing factors, such as the patient's vital signs, changes in the surgical process, etc. These factors make prediction of intraoperative anesthetic drug dosage more challenging. To address this challenge, we combine CNN and LSTM. CNN is able to extract local features in the input data, while LSTM is good at processing time series data and capturing long‐term dependencies in the data. By combining CNN and LSTM, we are able to make full use of various dynamic data during the intraoperative stage to achieve real‐time and accurate prediction of anesthetic drug dosage.

Based on the research of Nanaka et al., we improved the anesthetic drug dose prediction algorithm. Experimental results show that the method in this paper can obtain accurate anesthetic drug dose prediction, and is consistent with Nanaka et al.‘s view that anesthetic drug dose prediction methods based on machine learning or deep learning are effective.

To verify the effectiveness of the proposed model, we conducted a series of comparative experiments. We compared our model with traditional statistical methods and other deep learning models. The experimental results show that the method proposed in this study can effectively predict anesthetic dose. Compared to traditional methods, the anesthetic dose prediction error rate is reduced by 21.6% and the model fitting rate is increased by 16.1%, which is better than all comparative methods.

Additionally, we used the validation data set to perform specific experimental comparisons. As shown in Table 5, the proposed method demonstrates more accurate prediction results on the validation set compared to the traditional method through random sampling. These findings indicate that our model exhibits significant advantages in prediction accuracy, stability and real‐time performance, demonstrating the effectiveness and reliability of our model in anesthetic drug dose prediction.

**Table 5:** Comparison of test results on the validation set between traditional methods and the method proposed in this article.

Sex	Age	Weight	Systolic blood pressure	Diastolic blood pressure	Label	Anesthetic drugs etomidate	Muscle Relaxants ufentanil	Analgesics Atracurium	MSE
Female	55	59	110	60	TRUE	20	20	14	
					GRNN	18.12	23.35	10.58	8.82
					CNN	19.36	18.25	16.89	3.94
					LSTM	21.36	17.68	15.58	3.24
					ours	19.54	22.31	12.98	2.20
Male	34	70	127	86	TRUE	20	25	20	
					GRNN	19.83	19.8	18.63	9.65
					CNN	22.56	22.61	18.65	4.70
					LSTM	18.36	26.34	25.2	10.51
					ours	18.35	24.43	21.25	1.54
Male	61	79	130	82	TRUE	15	20	30	
					GRNN	17.65	18.28	22.68	21.19
					CNN	15.3	17.36	35.21	11.40
					LSTM	12.58	18.65	35.69	13.35
					ours	16.21	18.25	31.36	2.13

The prediction of anesthetic drug dosage using deep learning is a research field with significant application potential. By combining different types of neural network models, accurate predictions of anesthetic requirements can be achieved throughout surgical procedures. This not only enhances surgical safety and patient comfort, but also helps reduce medical resource waste and costs. In the future, we can explore advanced deep learning techniques to further optimize model performance and improve prediction accuracy. At the same time, we can also integrate more clinical data and expert knowledge into the model to better simulate the actual surgical environment and patient physiological changes.

Overall, the deep learning‐based approach to anesthetic drug dose prediction is a crucial area of study that can provide more reliable guidance for anesthesia management and contribute to improved patient outcomes and surgical success.

## 5 CONCLUSION

Based on various physiological information before and during surgery, this paper proposes a method based on deep learning to predict the dose of anesthetic drugs during the entire surgical process of the patient. The experimental results show that this method has a higher prediction accuracy of anesthetic drug dose, which basically meets the requirements of clinical conditions.

## AUTHOR CONTRIBUTIONS

**ZhiGang Hu**: Conceptualization; Formal analysis; Investigation; Methodology; Project administration; Supervision; Writing—review & editing. **GuangJian Pan**: Conceptualization; Data curation; Formal analysis; Investigation; Methodology; Software; Validation; Visualization; Writing—original draft; Writing—review & editing. **XinZheng Wang**: Conceptualization; Data curation; Formal analysis; Investigation; Methodology; Software; Validation; Visualization; Writing—original draft; Writing—review & editing. **KeHan Li**: Data curation; Formal analysis; Investigation; Resources; Validation. All authors have read and approved the final version of the manuscript.

## CONFLICT OF INTEREST STATEMENT

The authors have no conflicts of interest.

## ETHICS STATEMENT

1. We confirm that all methods were carried out in accordance with relevant guidelines and regulations.
2. We have been performed in accordance with the Declaration of Helsinki.
3. This original research was approved by the research Ethics Committee of The First Affiliated Hospital of Henan University of Science and Technology and did not require informed consent.

## TRANSPARENCY STATEMENT

The lead author XinZheng Wang affirms that this manuscript is an honest, accurate, and transparent account of the study being reported; that no important aspects of the study have been omitted; and that any discrepancies from the study as planned (and, if relevant, registered) have been explained.

## Data Availability

The data that support the findings of this study are available from the corresponding author upon reasonable request. CORRESPONDING AUTHOR or MANUSCRIPT GUARANTOR had full access to all of the data in this study and takes complete responsibility for the integrity of the data and the accuracy of the data analysis.
